# Exposure to Mercury and Aluminum in Early Life: Developmental Vulnerability as a Modifying Factor in Neurologic and Immunologic Effects

**DOI:** 10.3390/ijerph120201295

**Published:** 2015-01-23

**Authors:** José G. Dórea

**Affiliations:** Department of Nutrition, Faculty of Health Sciences, Universidade de Brasilia, 70919-970 DF Brasilia, Brazil; E-Mail: jg.dorea@gmail.com; Tel.: +55-61-8131-7800

**Keywords:** ethylmercury, methylmercury, aluminum, breastfeeding, Thimerosal, vaccines, neonates

## Abstract

Currently, ethylmercury (EtHg) and adjuvant-Al are the dominating interventional exposures encountered by fetuses, newborns, and infants due to immunization with Thimerosal-containing vaccines (TCVs). Despite their long use as active agents of medicines and fungicides, the safety levels of these substances have never been determined, either for animals or for adult humans—much less for fetuses, newborns, infants, and children. I reviewed the literature for papers reporting on outcomes associated with (a) multiple exposures and metabolism of EtHg and Al during early life; (b) physiological and metabolic characteristics of newborns, neonates, and infants relevant to xenobiotic exposure and effects; (c) neurobehavioral, immunological, and inflammatory reactions to Thimerosal and Al-adjuvants resulting from TCV exposure in infancy. Immunological and neurobehavioral effects of Thimerosal-EtHg and Al-adjuvants are not extraordinary; rather, these effects are easily detected in high and low income countries, with co-exposure to methylmercury (MeHg) or other neurotoxicants. Rigorous and replicable studies (in different animal species) have shown evidence of EtHg and Al toxicities. More research attention has been given to EtHg and findings have showed a solid link with neurotoxic effects in humans; however, the potential synergic effect of both toxic agents has not been properly studied. Therefore, early life exposure to both EtHg and Al deserves due consideration.

## 1. Introduction

The use of mercury (Hg) compounds in therapeutics goes back to the history of modern medicine. In the 18th century, Hg compounds used to treat syphilis were rubbed on to the skin of breastfeeding mothers, and this was reported to be an effective means of administering the therapeutic agent through breast milk [1]. Until relatively recently, there was a suite of medical products formulated to contain Hg as an active ingredient—mainly antiseptics and diuretics [2]. The use of Hg in pediatrics was broader and left a trail of toxic events of epidemic proportions; first described as “peculiar neurosis of the vegetative nervous system”, also known as acrodynia or “pink disease” [3]; the wide use of calomel in “worm cures and teething powders” [3] made it possible to track and tie acrodynia to Hg toxicity [4]. It is worth mentioning that topical Thimerosal used during pregnancy showed the highest relative risk of malformations compared to other non-mercurial antimicrobials [5]. Despite that, Thimerosal is still used in many pharmaceutical and hygienic products [6]. The continuing use of Thimerosal-containing vaccines (TCVs) in pediatrics (mostly during infancy) is still a matter of concern. In some developed countries, the use of TCVs is restricted to children six months or older. Thimerosal (used in multi-dose vials as an anti-microbial agent) was reduced in some pediatric vaccines in the USA, but it still remains in the majority of influenza vaccines and, with the influenza vaccine being added to the vaccine schedule, it continues to be given to pregnant women, infants, and children in the USA [7]. 

In most countries worldwide, because of costs, TCVs are recommended as safe [8] and used in pregnant mothers, newborns, neonates, and infants. The cost contention (between single-dose and multi-dose vials) has been recently challenged; Chhawchharia and Puliyel [9] argued that the use of multi-dose vials under actual conditions does not offer any real economic benefits. They showed studies reporting vaccine-wastage in multi-dose vials of up to 60%; thus, demonstrating that the price advantage of single-dose vials would require wastage no greater than 44%. Additionally, they contend that single-dose vials simplify inventory logistics and vaccine tracking as well as facilitate health workers to overcome reluctance to open multi-dose vials for few children, thus missing opportunity that could lead to lower coverage rates. 

Most TCVs are also adjuvanted with aluminum (Al). This type of adjuvant has been used in licensed vaccines for more than 70 years in different chemical forms (Al phosphate and Al hydroxide) and varying concentrations; the choice between the hydroxide or phosphate form depends on the electrostatic characteristics of the antigen (see references and discussion in Shaw *et al.*, 2013 [10]. Indeed, May *et al.* [11] reported that vaccines contain the highest concentrations of Hg and Al among all biological products (allergenic extracts, inactivated bacterial toxins including toxoids, blood, blood derivatives and others) tested. In the last 30 years, not only has the number of pediatric TCVs increased, but also the Al:Hg ratio, rising from around 10-12-fold [11] to 50-fold in some current vaccines that use Thimerosal at 0.01% [12,13,14]. Some hepatitis B vaccines (HBV) may contain additional phosphate to increase the phosphate-to-Al ratio in the Al hydroxyphosphate sulfate adjuvant, in order to potentiate its immunogenicity without increasing Al in the final product [15].

Although children are more susceptible than adults to toxic effects of heavy metal exposure, fetuses and neonates are even more vulnerable and the least protected by existing regulatory bodies [16]. Therefore, environmental and iatrogenic exposures to neurotoxic chemicals during critical periods of early life—in utero, neonatal and during infancy—are of particular concern. The proper function of the brain depends on the integrity of the whole central nervous system (CNS). Any developmental toxicity capable of affecting optimal development can have life-long consequences [17]; minimal dysfunction can negatively impact quality of life by increasing the risk of health problems and socioeconomic achievements [18]. Subtle neurochemical effects can have a profound and lasting influence on life trajectory [19].

In children, the toxicity of Hg compounds depends on the exposure mode (topical, oral, parenteral), dose (therapeutic, excipient in vaccines), duration (acute, chronic), developmental stage, be it prenatal or postnatal (neonates, infants, children, adolescent and adults); however, Hg exposure can occur simultaneously to more than one chemical form, along with other commonly co-occurring neurotoxic substances (Table 1). The most common environmental Hg exposure in early life is methylmercury (MeHg) through maternal fish-consumption (pregnancy and human milk); some Asian populations with high rice consumption are also exposed to MeHg. The most common interventional Hg exposure is through Thimerosal metabolite, *i.e.*, ethylmercury (EtHg), taken both by maternal immunization (during pregnancy) and directly by newborns and infants. In addition to specific issues related to physiological stages and unadjusted doses of EtHg, the bypassing of the gastrointestinal barriers by injected TCV-EtHg is expected to increase its toxicity [20]. During development of term and pre-term infants, partitioning of Hg compounds into tissues is expected to be different [20]. Distinct from maternal fish-MeHg exposure, food-Al in maternal diet is poorly (0.1% to 0.3%) absorbed and low amounts are transferred to milk [21]; it should be realized that the absorption of Al can be modified by dietary factors [22]. As a result, the maternal diet can increase the quantity of Al absorbed, which is expected to increase the concentration in the milk. As a corollary, the exposure of the suckling child is expected to be increased. Nevertheless, a breastfed child that is exposed to the regular schedule of TCVs is subjected to a larger load of parenteral Al adjuvants than that absorbed during nursing. Yet there are no studies of Al toxicity following immunizations with Al-adjuvanted vaccines; Shaw *et al.* [23] have reported that there might be differences in adverse events reported (from the vaccine adverse events report system) that could differentiate Al salts (potassium sulfate, hydroxide, and phosphate). A review of mechanisms of Al-adjuvant toxicity and autoimmunity in pediatric populations has been presented elsewhere [24]. 

Hg and Al share the ability to affect the neurological, renal, and immunological systems. Concentrations relevant to TCVs affect the developing CNS as demonstrated by *in vitro* and *in vivo* studies [25,26]. However, during the developmental stages of early life, the first (iatrogenic) encounter with xenobiotics, such as EtHg and Al-adjuvants, is a current feature of routine pediatric-immunization practices that need to be addressed.

## 2. Exposure and Metabolism of EtHg and Al During Early Life

During fetal development and post-natal stages, maternal exposure is the principal determinant of acquisition by the fetus and breastfed infant of Hg and Al [27,28]. Infant feeding practices can modulate the apportionment of environmental Hg and Al. While human milk has a steady concentration of these metals that depends on maternal exposure, formula feeding depends not only on the concentration of the elements in the chosen product but also on the concentration of these same elements in the water used to prepare the formulas [29]. Therefore, switching breast milk to formula, as a means of abating exposure, can, in some instances, increase the chance of higher exposure to mercury [29] and especially to aluminum in formulas based on cow’s milk or soya [30]. In either case, indirect (placenta and breast milk) or direct (TCVs) exposure poses special considerations.

### 2.1. Hg and Al Exposure in Human Milk, Infant Formulas, and in TCVs

During breastfeeding, the exposure to Hg and Al is proportional to the amount of colostrum or breast-milk consumed; different from a bolus dose in TCVs, breast milk is taken in proportion to infant size through various feeding periods throughout the entire lactation. Given the physiological differences in neonates to start and sustain breastfeeding, the most significant encounter of neonates with Hg and Al is through a TCV (HBV) given in the first 12 postnatal hours. In this specific vaccine, depending on the manufacturer, the Al (2500 µg):Hg (100 µg) ratio is 25 [12], whereas the 0.5ml dose for newborns will deliver a weight-adjusted dose of over a 20-fold variation among extremes of birth weight [31]. In either case, Hg or Al absorbed from colostrum by neonates (<24 h and weighing > 2000 g) can be far less than the loads inoculated through HBV; estimated loads of EtHg and Al in TCVs could attain a level corresponding to that absorbed from breast milk taken during the entire six months of lactation [28]*.* Indeed, the Al body burden from feeding (human milk and formulas) during the first year (0.1 mg) was estimated as much less than that (4 mg) attributed to vaccines [32]*.* Actually, this figure could increase, if we knew how much of EtHg and Al is transferred from TCVs during pregnancy [14].

In low and middle-income countries, vaccines preserved with Thimerosal continue to be administered to newborns (HBV), infants (HBV; diphtheria-tetanus-pertussis–DTP or the acellular variety DTaP), and young children (influenza 6 to 23 months (H. influenza type b-Hib, and meningococcal meningitis A/C/Y/W-135)). However, these are not the only exposures to TCVs; during pregnancy, mothers are immunized with the TCVs (diphtheria tetanus-DT, tetanus toxoid-TT, seasonal flu, and H1N1—swine flu). These vaccines are preserved with Thimerosal at a level of 0.01% and Al-adjuvant concentrations that vary according to type of vaccine [12].

EtHg is eliminated from infant blood more rapidly than MeHg [33], and blood biomarkers of exposure are, therefore, inherently less precise. As a consequence, compared with MeHg (which is retained in hair), a problem in epidemiological studies of non-persistent hair-EtHg is that imprecise assessment in non-invasive tissues makes exposure difficult to measure and obscures associations usually found for MeHg [14,34]. Human studies indicate that, once de-alkylated, the brain-retained Hg^2+^ species has a half-life of several years to decades following exposure [35]. These long residences in the brain clearly involve a long-lasting toxic effect. Efforts to track these sources of EtHg and Al in the hair of infants and young children have shown some promise for EtHg [36,37] but have been of very limited value for Al [38].

### 2.2. Physiological and Metabolic Characteristics of Newborns and Neonates Relevant to Xenobiotic Exposure and Effects

During early life, complex changes in the anatomical and chemical structure of the human body interact with xenobiotics in unique ways. Rapid organ development and function, along with changes in body composition, are related to cellular and metabolic activities that drastically affect distribution, organ uptake, and metabolism of xenobiotics [39,40]. These changes in early life are most prominent from the fetal stages up to the first six months; thus, variability becomes a key feature in limiting clearance capability, metabolism, and toxicity of xenobiotics; indeed, during the perinatal brain growth spurt and the formation of new connections between nervous cells makes the CNS especially vulnerable to toxic agents [41]. Therefore, assuming the dosing of drugs (and attendant effects) based purely on a body-weight basis can lead to erroneous conclusions [40,42]. In the case of neurotoxicity, this is further complicated by co-exposure (with positive and negative plasticity effects), exposure routes (oral and injected EtHg), and confounders related to prematurity and perinatal nurturing disturbance (interruption of breastfeeding). 

There are numerous health-promoting features of breastfeeding that might benefit infants in relation to both inorganic and organic Hg exposure and metabolism; notably, it reduces illnesses and, as a consequence, the use of antibiotics [43]. Breastfeeding is an important determinant of microbiotic colonization [44]. In this regard, due to changes in the intestinal microflora, antibiotics can impair demethylation of methylmercury and increase tissue retention of Hg [45]. The type of feeding after birth when the gastrointestinal tract starts functioning has the ability to modulate cysteine absorption through human casomorphin, thus regulating whole-body antioxidant capacity during early development [46,47]. Compared to older children and adults, neonates are deficient in the sulfate conjugation necessary to metabolize drugs taken by mothers during pregnancy and breastfeeding [48]. Premature neonates are also prone to low-albumin synthesis, a carrier protein essential to bind bilirubin, metals, and uremic toxins [49].

Considering that HBV is administered to premature babies, a 4.8-fold difference in TCV-EtHg and adjuvant-Al exposure exists between small and large neonates [28]. However, a difference in vaccine formulation regarding both preservatives (Thimerosal) and adjuvants makes a 20-fold difference in weight-corrected doses of EtHg [31]. Additionally, in the post-natal period, neonates lose up to 10% of body weight mainly as body water [27], accentuating metabolic differences between term and preterm babies [50] and between breastfed and formula-fed babies [51,52]. Furthermore, differences in the excretion (feces and urine pattern) of total Hg between newborns and infants [26] and blood Hg between stoolers and non-stoolers have been documented [53]. We also observed a difference between sexes regarding changes in body-Hg disposition (hair-Hg concentrations) during the neonatal period [54]. 

Metabolic stress related to food and nutrient intakes in animals has also been shown to accentuate Hg toxicity [26]; however, we do not know if other types of stress can influence the newborn’s ability to detoxify xenobiotics. It is still speculative if the amount of perinatal stress induced by types of delivery can influence the newborn to deal with detoxification mechanisms [55]. Nevertheless, Kapellou *et al.* [56] reviewed the importance of intrauterine brain development towards the end of pregnancy and possible effects on neurodevelopment that can occur if pregnancy is interrupted, such as premature delivery or interventional caesarean section. It is worth mentioning that stress associated with vaccination can also alter the metabolism of protein [57] and essential elements [58] in children. Additionally, these metabolic alterations can be compounded by the stressful irritability and aversion to feeding as side effects of HBV [59] and DTP [60] vaccines. It is also worth mentioning that preterm babies (mean 2254 g) exposed to an Al-adjuvanted vaccine did not show changes in serum and urine Al concentrations [58]. 

### 2.3. Insights Gained from Experimental Studies Modeling Early Life Exposure to TCVs

Existing (cell and whole animal) studies have demonstrated that Thimerosal/EtHg at concentrations found in vaccines is active against brain cells and can elicit biochemical and neurotoxic effects—including neurobehavioral outcomes; these studies are compatible with results obtained from other environmental Hg forms, such as MeHg in fish [61]. Developing humans (fetus > newborns > infants > toddlers > children) are more responsive to Hg exposure, thus needing study models that match such special characteristics.

Specific studies to address EtHg exposures compatible with vaccine schedules are recent and still very limited [62]. Animal studies (on monkeys, hamsters, mice, and two strains of rats) addressing biologic effects of TCV-EtHg have been discussed elsewhere [25]. Specific models demonstrating the neurodevelopment effects of EtHg doses simulating TCV on different species (mice, rats, and monkeys) have also been discussed elsewhere [26]; until then, there were no experimental studies modeling preterm (or small for gestational age) neonates. Recently, Chen *et al.* [63] modeled the effects in premature mice of vaccine doses of EtHg on “expression of dopamine D4 receptor (DRD4) and serotonin 2A receptor (5-HT2AR), apoptosis in the prefrontal cortex on post-injection day 49, and learning and memory functions.” A significant decrease in the expression of DRD4, 5-HT2AR, and learning function, and a significant increase in apoptosis were noted at the highest tested dose, while memory function was also significantly impaired at lower doses. Li *et al.* [64] showed in a neonatal mouse-model that EtHg is capable of inducing long-lasting substantial dysregulation of neurodevelopment, synaptic functions, and endocrine systems; in this animal model, males are more susceptible to Thimerosal-Hg toxicity than females [65]. Clear differences in the brain:blood ratio have been estimated as 0.06, 1.2, 6, and 6 in rats, mice, squirrel monkeys, and humans respectively [66]. 

In experimental studies, the half-life of Thimerosal-Hg is higher in the brains than in the blood of mice [67]. Immature mice (10 days postnatal), given single intramuscular doses of Thimerosal, showed relatively lower organic mercury levels in the brain but relatively higher inorganic Hg concentrations when compared with MeHg-treated animals [68]. Acute exposure to Thimerosal and EtHg revealed that the brain distribution was respectively much higher (0.22% and 0.4%) in immature mice (postnatal day 16) than in adult (0.22% and 0.4%) animals [69]. Actually, mice models demonstrating neurotoxic effects of TCV-Hg are conservative in relation to both EtHg and Al doses relevant to newborns [70]. In primates (*Macaca fascicularis*), brain Hg after exposure (at birth, one, two, and three weeks postnatal) to TCV produced brain total-Hg concentrations paralleling blood half-lives [71]. Dose translation of EtHg from different animal species to developing humans is rather complex and it may need to consider an equivalent dose that takes into consideration body surface area and not only just body weight [72]. Furthermore, current animal studies are inadequate to provide information on EtHg and brain acute toxicity.

Regarding Al, Veiga *et al.* [73] showed the greatest Al accumulation in all tissues of newborn rats (including the cortex and hippocampus) compared to older age groups receiving intraperitoneal doses of Al. In mice, an injection of a vaccine containing Al showed extremely slow clearance from the CNS [74]. Recent animal experiments with Al concentrations relevant to vaccines have suggested adverse neurological effects [75]. Genetic background or prior history of adverse reactions to vaccines may predispose individuals to neurological adverse effects provoked by Al; in animals modeling autoimmune disease (systemic lupus erythematosus), immunization with Al affected blood counts, neurocognitive functions and brain gliosis [76]. Therefore, the need for *in vitro* experimental models designed to assess the isolated and combined effects of EtHg and Al^3+^ is clear. Although they cannot substitute the *in vivo* experiments, they can be used to rationalize *in vivo* studies. 

Therefore, experimental neurotoxicity studies modeling interventional exposure to EtHg and Al are still in their infancy, but they are crucial in guiding protective measures regarding TCVs (EtHg and Al) used in developing infants. These studies are needed in relation to constitutional diversity, environmental co-exposures and other modifying factors. Existing animal studies, so far, have consistently shown the effects of Thimerosal/EtHg on neurobehavioral responses at exposure levels compatible with those encountered in pediatric vaccines [26]; Thimerosal/EtHg failed all animal studies [26]. 

## 3. Are TCV-EtHg and Al-Adjuvant Exposures in Early Life Associated with Neurodevelopment Outcomes in Childhood?

Despite the long history of tragic accidents with organic Hg (which included EtHg), studies addressing the potential link of TCV-EtHg and neurodevelopment started only after Thimerosal was withdrawn from pediatric vaccines in high income countries. Large retrospective studies to assess the impact of TCV on immunological and neurodevelopment appeared only in the last 10 years [77]. Although the most controversial part of TCV was its putative link to autism, this so far has no known single established cause; a review of studies addressing only Thimerosal exposure individually and neurodevelopment (and not an autism diagnostic) is found elsewhere [77]. Before such studies, the risk of neurotoxicity associated with TCVs was estimated as plausible [78].

Studies of neurodevelopment outcomes that do not include autism can be grouped into two types—those that assessed TCV-EtHg as a single exposure variable and studies that included TCV-EtHg in a model of multiple exposure (to neurotoxic variables) related to parental lifestyle (smoking), food habits (maternal fish and rice consumption) or amalgam fillings (Table 1). 

The studies that considered TCV-EtHg as a single exposure evaluated neurodevelopment retrospectively (five studies); these studies explored passive data banks from the UK and the USA and used different statistical strategies [77]. Along with three more prospective studies, they showed that despite ambiguity, the risk of neurotoxicity is plausible at least for susceptible infants [78].

In real-life scenarios, a developing fetus or infant can be exposed to environmental challenges that may impact neurodevelopment. When TCV is part of a statistical model that reflects real-life challenges with more than one neurotoxic exposure (confounded with positive and negative neuroplasticity), the results were less ambiguous (Table 1) than those reviewed elsewhere [77]. Children with full breastfeeding for at least 6months and with an equal vaccination scheme showed no significant association between TCV and neurodevelopment measured by the Gesell Developmental Scores (GDS) (Table 1); also, in cross-sectional studies, when GDS were evaluated over a wide age range (0–5 years), there were no significant associations (Table 1). However, in multivariate analysis, studies that controlled some factors known to influence neuroplasticity consistently suggested a negative interaction between EtHg and neurodevelopment: in Brazil, controlled variables were age of Gesell evaluation and sources of environmental neurotoxic exposure [14,34,79]; in Korea, the controlled variable was secondhand smoke [80]; and in Poland, controlled variables were secondhand smoking, cord-blood Hg and Pb [81,82]. 

Studies in children (from France, the Czech Republic, and Poland) conclude that we do not have a safe value of concentration showing a clear threshold for renal and neurologic biomarkers of damage caused by Hg [83]; it is therefore even more difficult to determine exposure levels that may cause neurodevelopmental harm. Therefore, it is not possible to expect robust statistically significant effects from small-sized studies (Table 1). However, in studies with a larger sample and longitudinal design, the consistent trend (most of the studies showing significant associations) indicates that the inference of neurotoxicity due to Thimerosal in vaccines is sustainable and science-based (Table 1). Therefore, regarding mercury neurotoxicity in children, the combined evidence of experimental [25] and population studies (Table 1) is strong and credible; regarding mercury toxicity in pediatric populations, it is not possible to wait for the perfect data [84]. Grandjean and Herz [85] have discussed the impact of uncertainties related to low doses of organic Hg during brain development and underestimation of neurotoxicity. Therefore, it is arguable that the extensive use of pediatric TCV-EtHg delivered over a wide range of weight-unadjusted concentrations in neonates is without consequences [31]. 

In addition to the multiple exposure studies in Table 1, a number of epidemiological studies found significant dose dependent relationships between Thimerosal administration and medically diagnosed specific delays in development [86,87,88,89]. Also Gallagher and Goodman [90] reported a significant association between TCV exposure from hepatitis B vaccine and developmental disability in U.S. children aged one to nine years. By contrast, other investigators have failed to find a consistent significant relationship between TCV exposure and subsequent specific delays in development in exposed children [91,92]. However, those studies were recently reviewed and it was concluded that their results were uninterpretable [93].

## 4. Immunological and Inflammatory Reactions to Thimerosal and Al-Adjuvants Resulting from TCV Exposure in Infancy

Children are more sensitive than adults in reacting to Thimerosal patch-test and contact allergies. Indeed, the pattern for a positive patch-test to Thimerosal has changed with the use (or discontinuation) of TCV in children. Countries that withdrew Thimerosal from pediatric vaccines, such as Austria, Denmark, Poland, Greece, and the USA, observed a decrease in Thimerosal patch-test reactions in tested populations [94]. Thimerosal patch-test reactions from vaccines are considered of no clinical significance. However, Kravchenko *et al.* [95] speculated that Thimerosal can kill and damage the cells at the site of the injection, thus changing their properties, and that it is also capable of inducing the formation of autoantigens with unpredicted results. These same speculations have been developed regarding exposure to Al at the injection site [10,96].

While the use of TCVs and Thimerosal-free vaccines showed differences in atopic dermatitis, a comparison of reactions between Al hydroxide and Al phosphate seems to elicit different responses to adjuvanted-Al adverse reactions. Shaw *et al.* [10] observed that Al phosphate favors a systemic reaction, whereas Al hydroxide favors a local reaction; they hypothesized that a difference in Al charges can favor binding to membrane sulfates at the site of the injection (Al hydroxide) or be freer (Al phosphate) to move and migrate to other tissues [10]. In rabbits, Hem *et al.* [97] showed differences in Al-adjuvant body retention, depending on its chemical form; there was three times more Al absorbed from aluminum phosphate than from aluminum hydroxide.

We have few studies addressing the exposure of Al adjuvant used in pediatric vaccines. In adults, macrophagic myofasciitis has been reported as a result of Al-adjuvanted vaccines [98]. This uncommon inflammatory myopathy has been reported in children [99]. Besides the development of hypotonia and psychomotor delay, the observed histologic pattern was not associated with a distinctive clinical syndrome [99]. An apparently under-reported allergy described as persistent itching subcutaneous nodules is caused by Al-adjuvanted vaccines [100]. Although Al phosphate is considered less reactogenic than Al hydroxide [101], in this series of patients, no difference was attributed to the Al chemical form (hydroxide or phosphate) used in the European vaccines [100,101]; it was reported that children that developed itching granulomas also developed contact allergic to Al. 

Lisciandro *et al.* [102] reported differences in age-related innate-immune responses to Al from vaccines at different ages during infancy that depended on the ligands and cytokines. Whole blood innate immune responses to the Al (vaccine adjuvant) decrease with age. They also suggested that in populations experiencing higher infectious pressure, there could be different patterns of innate immune development; thus clearly implying differences between geographically diverse populations [102]. More recently, Terhune and Deth [103] reviewed impaired regulatory T cell function as an adverse response to Al-adjuvanted vaccines in genetically susceptible individuals. Al body burden potential toxicity resulting from immunotherapy has been reviewed elsewhere [104].

**Table 1:** Chronologically organized summary of studies addressing multiple exposures that include Thimerosal-containing vaccines (TCVs) given to infants and neurodevelopment outcomes, by country, age, neurological test used, and the co-occurring exposure.

Reference	Country	n	Age of Test	Test	Additional Exposure to TCV	Outcomes
Marques *et al.* [105]	Brazil	82	6 m	GDS	Reanalysis of 23 variables that included maternal and infant exposure analyzed by a mathematical model (PCA).	Principal Component Analysis discriminated variability of early vaccine schedule and neurodevelopment outcomes associated with variables that included pre- and post-natal Hg exposure.
Marques *et al.* [106]	Brazil	82	6; 36; 60 m	GDS	Prenatal maternal exposure to MeHg.	No significant association with day of the first postnatal dose of a TCV (HBV).
Marques *et al.* [107]	Brazil	82	6 m	GDS	Prenatal maternal exposure to TCVs and MeHg.	GDS at 6 m was significantly associated with total Hg of neonate’s hair but was not sensitive to the number of TCVs taken by the mother.
Marques *et al.* [108]	Brazil	249	0 to 5 y	GDS	MeHg (HHg) in children of traditional fish eaters.	No significant association with total TCV-EtHg exposure at time of test.
Marques *et al.* [109]	Brazil	688	0 to 5 y	GDS	Hair-Hg concentrations in children of tin-ore miners.	No significant association with total TCV-EtHg exposure at time of test.
Dórea *et al.* [79]	Brazil	281	6 m	GDS	Prenatal maternal exposure to MeHg.	A higher score of neurological development at six months was negatively associated with exposure to additional TCV-EtHg.
Lee and Ha [80]	South Korea	299	6 m	BSID-II	Second hand smoking.	There were marginal differences in MDI scores according to TCV history (information incomplete).
Mrozek-Budzyn *et al*. [81]	Poland	196	12; 24; 36 m.	BSID-II	Second hand smoking; cord blood-Hg; cord blood-Pb.	An adverse effect of neonatal TCV exposure was observed for the PDI only in the 12th and 24th months of life.
Marques *et al.* [14]	Brazil	96	6; 24 m	BSID-II	Prenatal maternal exposure to MeHg and environmental Pb.	MDI and PDI were statistically significant (respectively *p* < 0.0000001, *p* = 0.000007) lower for the children living in a multi-exposure environment that included higher EtH exposure only at 24 months of age. Multivariate regression analysis showed that MDI was negatively affected by breast-milk Pb and by HHg. PDI was positively affected by breastfeeding and negatively affected by EtHg.
Dórea *et al.* [34]	Brazil	299	12 to 24 m	GDS	Hair-Hg concentrations in children of tin-ore miners and fishing villages.	Despite significantly higher exposure to both forms of organic Hg (MeHg from maternal fish consumption, and EtHg from TCV) in toddlers from the fishing village, significant differences were seen only among the proportions of most severely affected toddlers (GDS *<* 70).
Mrozek-Budzyn *et al*. [82]	Poland	318	6 m. 12; 24; 36 m. 6;7;9 y	Fagan BSID-II WISC	Second hand smoking; cord blood-Hg; cord blood-Pb.	Adverse effects on cognitive tests (Fagan, MDI only at 36 month, and WISC only at 9 y) were observed for neonatal TCV exposure.
Marques *et al.* [110]	Brazil	294	6; 24 m	BSID-II	Prenatal maternal exposure to MeHg in tin-ore mining settlement.	No significant association of BSID with total TCV-EtHg exposure at time of test. There was a significant sex difference in neurodevelopment, with boys showing more sensitivity related to BSID delays.

BSID-II: The Bayley Scales of Infant Development, second edition; EtHg: ethylmercury; GDS: Gesell Development Scores; HBV: hepatitis B vaccine; MDI: Mental Development Index; MeHg: methylmercury; m: months; PDI: Psychomotor Development Index; y: years; WISC-R: Wechsler Intelligence Scale for Children-Revised.

## 5. Conclusions

- Newborns vary in size, organ development, genetics, pregnancy environment; these characteristics *per se* could slow metabolism or accentuate toxicity of xenobiotics, yet fetuses and infants are continually exposed to EtHg doses proven to produce effects on experimental models.
- The uptake and elimination rates of Hg during the neonatal period (especially in preterms and small-for-gestational-age newborns) are different from anything experienced in later stages of development or during adulthood.
- In the most vulnerable period of human development, Hg and Al transfer-efficiencies into brain tissues and assessment of neurochemical effects are difficult to model and also to interpret functional outcomes in older ages.
- Observational and cohort studies have consistently shown significant interactions of TCV-EtHg compatible with Hg toxicity at low doses.
- We need models relevant to pediatric vaccines to test early EtHg and Al exposures in relation to constitutional and environmental co-exposures and other modifying factors.
- Concerns about the safety of Thimerosal (in relation to the developing CNS) are undervalued relative to its use as a preservative in pediatric vaccines on account of costs.
- Any interventional agent should respect the special stages of the developing human brain—in fetuses, infants, and young children. Therefore, specific recommendations for newborns and preterms should be in place for immunization with TCVs.

Summing up: Rigorous and replicable studies (in different animal species) have shown evidence of EtHg, and of Al toxicities. More research attention has been given to EtHg and findings have showed a solid link with neurotoxic effects in humans; however, the potential synergic effect of both toxic agents has not been properly studied. Therefore, early life exposure to both EtHg and Al deserves due consideration.
